# BCG after trimodality therapy: managing non-muscle-invasive recurrence in the irradiated bladder

**DOI:** 10.3389/fonc.2026.1953755

**Published:** 2026-09-01

**Authors:** Nicola Fazaa, Etan Eigner, Melissa Atallah, Yuval Shafran, Gilad Amiel, Azik Hoffman

**Affiliations:** Department of Urology, Rambam Health Care Campus, Haifa, Israel

**Keywords:** Bacillus Calmette–Guérin, bladder cancer, bladder preservation, intravesical therapy, non-muscle-invasive bladder cancer, salvage cystectomy, trimodality therapy

## Abstract

Trimodality therapy (TMT) is an established bladder-preserving option for selected patients with muscle-invasive bladder cancer, but non-muscle-invasive recurrence remains a clinically relevant challenge. The role of intravesical bacillus Calmette–Guérin (BCG) after prior bladder-directed chemoradiation is uncertain because the available evidence is limited to small retrospective cohorts and indirect studies in previously irradiated bladders. This mini-review examines the efficacy and tolerability of BCG after TMT, the uncertain role of maintenance therapy, and the factors that should guide continued bladder preservation versus salvage cystectomy. Across available post-TMT series, BCG was feasible and durable disease control occurred in some patients; however, the small, highly selected cohorts are vulnerable to selection bias and limited statistical power, precluding definitive comparative claims or definition of an optimal induction or maintenance strategy. Careful staging is essential because radiation-related changes may complicate endoscopic assessment and pathologic interpretation. BCG may be considered when recurrence is clearly non-muscle-invasive, endoscopically manageable, and the bladder remains functional, with salvage cystectomy still feasible if treatment fails. Uncertain staging, incomplete resection, persistent or early recurrent high-risk disease, BCG-unresponsive recurrence, or poor bladder function should lower the threshold for cystectomy. Prospective studies should incorporate oncologic outcomes, treatment completion, urinary function, and cystectomy-free survival.

## Introduction

Radical cystectomy remains a standard curative treatment for muscle-invasive bladder cancer, but its benefits must be weighed against substantial perioperative morbidity, the need for urinary diversion, and potential long-term effects on function and quality of life (1–3). For appropriately selected patients, bladder-preserving trimodality therapy (TMT) offers an alternative, including for those who are medically unsuitable for cystectomy or prefer bladder preservation (1, 4). TMT combines maximal transurethral resection of the bladder tumor (TURBT) with concurrent chemoradiotherapy (1, 4). Contemporary series have reported favorable long-term oncologic outcomes, with native-bladder preservation rates approaching 85% at 5 years (4, 5).

Preserving the native bladder requires continued surveillance, as non-muscle-invasive bladder cancer (NMIBC) recurrence occurs in approximately 20–26% of patients after TMT (4, 6, 7). Muscle-invasive relapse is generally viewed as failure of bladder preservation and usually leads to salvage radical cystectomy in surgically fit patients (8). By contrast, NMIBC recurrence may remain amenable to further bladder-sparing management. Low-grade Ta tumors can often be treated endoscopically, whereas high-grade Ta, carcinoma *in situ* (CIS), or T1 disease raises a more difficult choice between repeat TURBT with intravesical therapy and early cystectomy (9, 10).

Although intravesical BCG is well established in the management of intermediate- and high-risk primary NMIBC (11), its role after prior bladder chemoradiation is less straightforward (9, 12). Treatment decisions must account for the challenges of staging recurrent disease in an irradiated bladder, the functional consequences of further intravesical therapy, and the risk of delaying potentially curative salvage cystectomy. The available evidence is limited and is derived predominantly from small, retrospective, and highly selected cohorts (12). This mini-review examines the efficacy and tolerability of BCG after TMT, the uncertain role of maintenance therapy, and the clinical factors that may guide continued bladder preservation versus salvage cystectomy. Whereas prior reviews have addressed recurrence management after TMT more broadly (9), the present review focuses specifically on BCG efficacy and tolerability, the uncertainty surrounding maintenance, and a practical decision framework for continued bladder preservation versus salvage cystectomy.

### The post-TMT bladder: diagnostic, functional, and biological considerations

Before intravesical therapy is considered, recurrent disease must be confirmed and accurately staged. In the irradiated bladder, erythema, telangiectasia, ulceration, friability, necrotic debris, and chronic inflammation can mimic tumor or obscure flat carcinoma *in situ* (CIS), while fibrosis and impaired healing may complicate complete resection and pathologic staging (13–15). In the absence of post-TMT-specific staging data, evaluation should generally follow established NMIBC principles: visible lesions should undergo complete TURBT or targeted biopsy, the resection specimen should contain detrusor muscle when invasion is suspected, and repeat TURBT should be performed for T1 disease, incomplete resection, or absence of detrusor muscle when staging remains uncertain (16). In patients with positive cytology and no visible lesion, mapping or photodynamic diagnosis-guided biopsies should be used selectively, together with evaluation of the upper urinary tract and prostatic urethra (16).

Accurate staging alone does not determine whether further bladder preservation is worthwhile. Chemoradiation may leave patients with persistent storage symptoms, hematuria, pain, reduced capacity, or impaired compliance, all of which may be aggravated by repeated BCG instillations (17). Baseline urinary function and bladder capacity should be documented before treatment; a bladder that is preserved anatomically but causes severe symptoms or has poor capacity may offer little clinical benefit. The assessment should also consider whether the patient remains fit for salvage cystectomy if intravesical treatment fails.

Post-TMT recurrence may not be biologically equivalent to *de novo* NMIBC. In a genomic analysis of chemoradiation-treated urothelial carcinoma, five matched primary-recurrence pairs shared a median of 92% of somatic mutations, supporting the hypothesis that some recurrences may arise from pre-existing treatment-resistant clones rather than from a biologically new tumor (18). The study did not evaluate BCG response, and its small matched cohort precludes broader conclusions. At present, it remains unknown whether radiation-induced changes in the urothelium, stroma, or immune microenvironment alter the efficacy of intravesical BCG.

### Clinical evidence for BCG after bladder-preserving chemoradiation

The direct evidence for BCG after TMT is limited to retrospective studies with small treated cohorts and substantial treatment selection based on tumor characteristics, bladder function, and physician judgment (12, 19, 20). The available reports also differ in design: one describes BCG outcomes within a larger post-TMT recurrence cohort, one evaluates conservative management against matched *de novo* NMIBC controls, and one directly compares BCG-treated patients with and without prior bladder-sparing therapy (19, 20).

Sanchez et al. retrospectively reviewed 342 patients who achieved a complete response after TMT. NMIBC recurrence developed in 85 patients (25%); 39 received intravesical BCG, of whom 29 completed induction therapy. Following induction BCG, estimated 3-year recurrence-free and progression-free survival were 59% and 63%, respectively. Treatment was not assigned prospectively, and the study lacked a non-irradiated BCG control; the reported outcomes therefore document activity within this cohort but do not establish comparative efficacy (12).

Ajib et al. compared 12 patients with NMIBC recurrence after TMT with 60 *de novo* NMIBC controls matched by stage and grade. Post-TMT recurrences comprised Ta disease in four patients, T1 disease in three, and CIS in five. Subsequent recurrence occurred in 2 of 12 post-TMT patients (17%) and 38 of 60 controls (63%), while cystectomy was performed in 1 of 12 (8%) and 11 of 60 patients (18%), respectively. The post-TMT cohort was older and had shorter follow-up than the control group, and treatment was not limited to BCG. The study therefore supports the feasibility of conventional NMIBC management after TMT but cannot determine the independent effect of BCG (19).

The most direct comparative evidence comes from the retrospective study by Du et al., which specifically evaluated BCG after NMIBC recurrence following bladder-sparing treatment for MIBC. Five-year recurrence-free survival was 66.7% in the post-MIBC group and 57.0% in the conventional NMIBC group; corresponding overall survival estimates were 75.8% and 87.4%. No statistically significant differences were detected in the survival analyses (20). However, the 19-patient post-MIBC cohort provided limited statistical power, and the absence of significant differences should not be interpreted as evidence of equivalence. The study nevertheless provides the most direct comparative data currently available (20).

Indirect evidence comes from patients treated with BCG after prostate radiotherapy. In a SEER-Medicare analysis of 3,466 men who received adequate BCG for high-risk NMIBC, including 145 with prior prostate radiotherapy, previous irradiation was not associated with the 5-year composite risk of progression after multivariable adjustment (adjusted hazard ratio 0.99; 95% CI, 0.61–1.58) (21). In a separate 26-patient retrospective series, 13 patients (50%) were successfully managed with one or more BCG-based induction courses at a mean follow-up of nearly 5 years (22). These studies evaluated oncologic outcomes rather than local toxicity or treatment adherence, and prostate radiotherapy is not equivalent to bladder-directed chemoradiation. Important differences in radiation target and dose distribution, as well as the use of concurrent radiosensitizing chemotherapy in TMT, further limit direct extrapolation to the post-TMT setting. Their relevance to the post-TMT population is therefore indirect.

In these reports, BCG was feasible after TMT, and durable disease control was observed in some patients. The data are too limited to establish equivalence with primary NMIBC, identify who is most likely to benefit, or determine the optimal induction and maintenance strategy (12, 19, 20).

### BCG tolerability and maintenance in the irradiated bladder

BCG commonly causes local urinary symptoms and can occasionally produce systemic toxicity; treatment discontinuation occurs even in non-irradiated NMIBC populations (23). After bladder-directed chemoradiation, toxicity may compound pre-existing urinary morbidity. Relevant measures of tolerability therefore include completion of induction, exposure to maintenance, and whether bladder function remains acceptable during treatment.

The limited clinical data on BCG after bladder irradiation are reassuring, although clearly selected. In the large post-TMT NMIBC recurrence cohort, 39 patients were treated with TURBT followed by intravesical BCG; 29 completed induction therapy, while BCG-related toxicity was reported in 19 patients (12). A more recent BCG-specific retrospective series provides more granular safety data. Among patients treated for NMIBC recurrence after prior bladder-sparing therapy, adverse events were reported in 89.5%, nearly identical to the 89.0% rate observed in conventional intermediate- or high-risk NMIBC controls. Urgency or frequency was common in both groups (89.5% vs. 86.1%), while dysuria (21.1% vs. 32.7%), hematuria (15.8% vs. 20.8%), bladder contracture (5.3% vs. 4.0%), and mycobacterial complications (5.3% vs. 1.0%) were not increased in the post-bladder-sparing cohort. Fever and urinary retention were also less frequent in this group (20). These data do not eliminate concern about tolerability in the irradiated bladder, but they argue against viewing prior bladder irradiation as a contraindication to BCG when patients are carefully selected.

Maintenance remains the least settled part of BCG treatment after bladder irradiation. Existing post-TMT studies provide evidence that induction can be completed in some patients, but they do not define whether maintenance improves outcomes or which schedule should be used. In a retrospective cohort of 723 patients with conventional NMIBC, discontinuation because of toxicity occurred in 24% of patients receiving monthly maintenance and 28% of those treated according to the SWOG schedule (24). This study provides general context regarding maintenance tolerability but did not include a post-TMT population. In the Du et al. study, patients treated after bladder-sparing therapy for MIBC received fewer BCG instillations than conventional NMIBC controls (9 vs. 15.5; P = 0.010), but the reason for this difference was not established (20). The available post-TMT reports provide insufficient detail regarding dose reductions, schedule modifications, or the reasons for lower total BCG exposure. It should not be interpreted as evidence that reduced maintenance is equally effective or that post-TMT tumors are more responsive to BCG. The optimal maintenance regimen after TMT remains unknown. Decisions should account for treatment tolerance, bladder function, response to induction, and patient preference; abbreviated maintenance has not been shown to provide equivalent oncologic control in this population.

### Patient selection and the threshold for salvage cystectomy

After TMT, the first management distinction is between non-muscle-invasive recurrence and muscle-invasive relapse. Muscle-invasive recurrence should prompt evaluation for salvage radical cystectomy in surgically fit patients, whereas NMIBC may remain amenable to further bladder-directed treatment (25). BCG should be considered only when staging is reliable, the disease is endoscopically manageable, and bladder function remains sufficient to justify preservation.

By extrapolation from standard high-risk NMIBC management and the limited post-TMT series, potential candidates include completely resected high-grade Ta tumors, CIS without evidence of invasive disease, and selected T1 recurrences after adequate restaging with repeat TURBT (11). Small low-grade Ta recurrences generally do not require BCG and can usually be managed endoscopically, with or without intravesical chemotherapy (25).

The threshold for salvage cystectomy should depend less on the label of NMIBC recurrence than on staging confidence and the likelihood of durable control with intravesical therapy. Post-TMT series include Ta, CIS, and T1 recurrences managed without immediate cystectomy, but their retrospective design and treatment selection do not define which tumors can safely undergo repeated bladder-sparing treatment (12, 19). Evidence from conventional high-risk NMIBC is relevant but indirect. In a retrospective cohort of 303 BCG-treated patients with pT1 high-grade disease, extensive or multifocal lamina propria invasion was independently associated with progression (HR 5.3; 95% CI, 2.2–13.1) (26). In a separate multicenter cohort of 578 patients with BCG-unresponsive NMIBC, initial bladder-sparing treatment was not associated with inferior intermediate-term survival, but high-grade recurrence and progression increased over time, and nodal disease was more frequent at later than upfront cystectomy (13% vs. 4%) (25, 26). These studies do not define a post-TMT threshold, but they support early cystectomy discussion when staging is uncertain, complete resection is not feasible, T1 invasion is extensive or multifocal, CIS persists, high-grade recurrence is early or extensive, or disease becomes BCG-unresponsive (25, 26).

**Figure 1:**
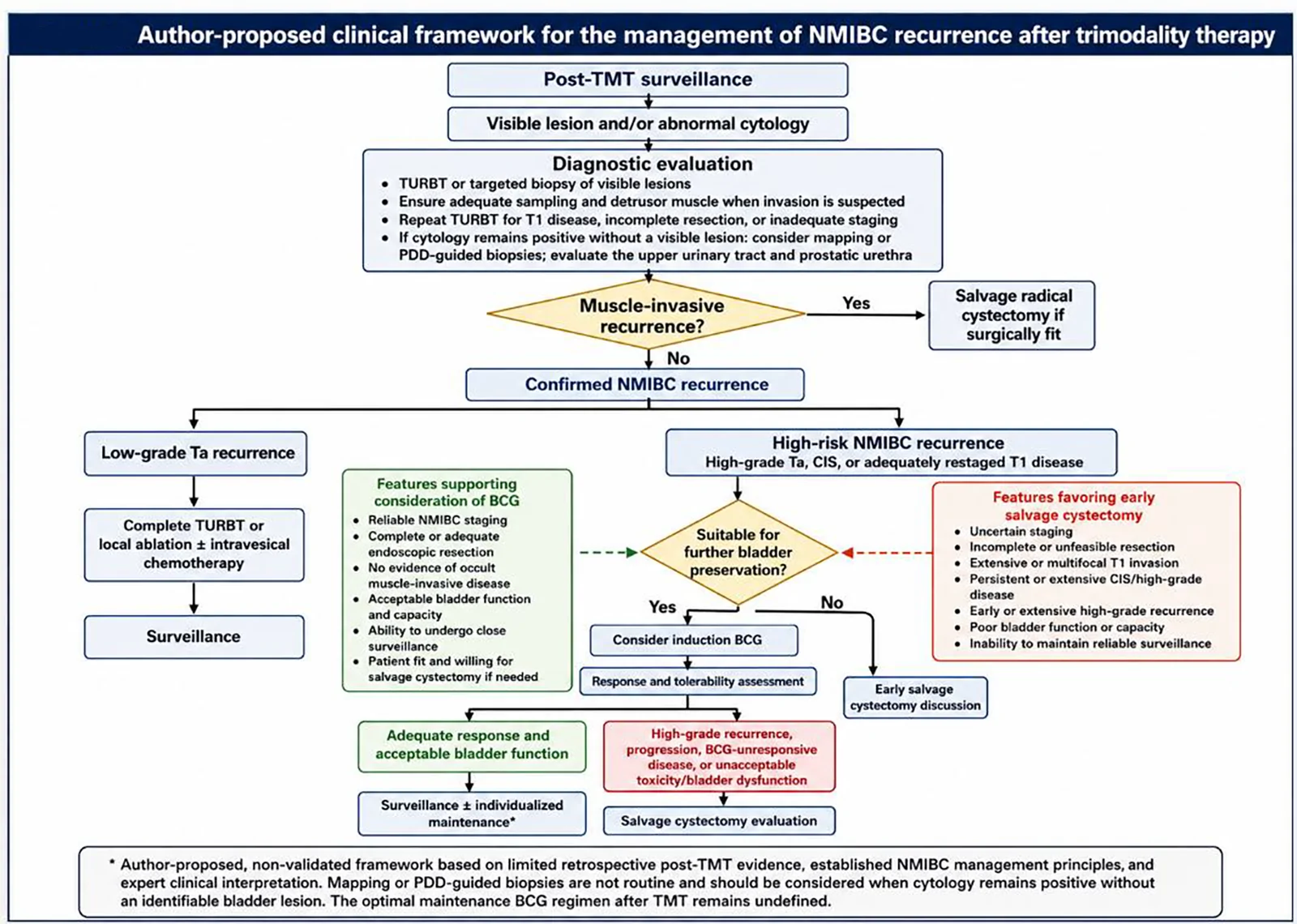
Author-proposed clinical framework for the management of non-muscle-invasive bladder cancer recurrence after trimodality therapy. The framework is based on limited retrospective post-TMT evidence, established NMIBC management principles, and expert clinical interpretation, and has not been prospectively validated. Mapping or photodynamic diagnosis-guided biopsies are not recommended routinely and should be considered when cytology remains positive without an identifiable bladder lesion. The optimal maintenance BCG regimen after TMT remains undefined.

Before BCG is started, the patient’s fitness and willingness to undergo salvage cystectomy if treatment fails should be established. **Figure 1** presents an author-proposed, non-validated framework integrating tumor stage, resectability, recurrence pattern, bladder function, and surgical fitness.

## Discussion

Available evidence indicates that intravesical BCG can be administered after TMT and may provide durable control in a proportion of patients with NMIBC recurrence. However, the evidence is derived from small retrospective post-TMT cohorts and indirect studies of BCG after prostate radiotherapy. These data establish neither equivalent efficacy nor equivalent tolerability compared with primary NMIBC (12, 19–22).

The clinical decision is therefore not based on prior irradiation alone. BCG may be considered when recurrence is confidently staged as non-muscle-invasive, complete endoscopic management is feasible, bladder function remains acceptable, and timely salvage cystectomy remains an option if treatment fails. Uncertain staging, incomplete resection, persistent or early recurrent high-risk disease, BCG-unresponsive recurrence, or poor bladder function should lower the threshold for cystectomy. The optimal role of maintenance after TMT remains undefined, and abbreviated treatment has not been shown to provide equivalent oncologic control.

Future studies should report not only recurrence and progression, but also induction completion, maintenance exposure, treatment discontinuation, patient-reported urinary function, cystectomy-free survival, and outcomes after salvage cystectomy. Treatment sequencing after BCG failure also remains unresolved. Newer options for BCG-unresponsive NMIBC, including nadofaragene firadenovec and intravesical gemcitabine delivery systems such as TAR-200 (27),have not been specifically studied in the previously irradiated post-TMT bladder, and their efficacy and tolerability in this setting remain unknown.

BCG can be considered for adequately staged NMIBC recurrence after TMT when the tumor is endoscopically manageable and the bladder remains functional. This approach requires close surveillance and readiness to proceed to salvage cystectomy when invasive relapse, BCG-unresponsive disease, or unacceptable bladder dysfunction develops. Given the small, highly selected retrospective cohorts and limited statistical power, these findings should be interpreted as evidence of feasibility rather than comparative equivalence. Treatment decisions should remain individualized and should be discussed within a multidisciplinary bladder cancer team.
